# Congenital Thrombotic Thrombocytopenic Purpura Presenting as Recurrent Ischemic Stroke in a Young Adult: A Genetically Confirmed Case

**DOI:** 10.7759/cureus.109399

**Published:** 2026-05-21

**Authors:** Rajashekar V, Mukil Soundar, Senthilpriyan K, Akshaya Kumar Balagopal, Hema K, Vyas R, Santhi T S

**Affiliations:** 1 Internal Medicine, Madras Medical College, Chennai, IND; 2 General Medicine, Madras Medical College, Chennai, IND

**Keywords:** renal insufficiency, stroke, thrombotic microangiopathies, thrombotic thrombocytopenic purpura, young stroke

## Abstract

Thrombotic thrombocytopenic purpura (TTP) is a rare thrombotic microangiopathy that can present with neurological and renal involvement and may rarely manifest as acute ischemic stroke in young adults. We report the case of a 23-year-old male who presented with acute-onset right-sided hemiplegia and expressive aphasia, with a history of recurrent transient ischemic attacks and renal dysfunction. Laboratory evaluation revealed severe thrombocytopenia, microangiopathic hemolytic anemia with schistocytes, and markedly elevated renal parameters. Neuroimaging demonstrated an acute infarct predominantly involving the left parietal region with additional subacute infarcts, while intracranial vessels were normal. A PLASMIC (Platelet count, combined Hemolysis parameters, Absence of active cancer, no history of solid Organ or Stem cell transplant, MCV (mean corpuscular volume), INR (international normalized ratio), and Creatinine) score of 5 indicated an intermediate risk for severe ADAMTS13 deficiency. The patient was treated with high-dose corticosteroids during the acute phase along with supportive therapy. Genetic testing subsequently confirmed congenital TTP due to a homozygous ADAMTS13 mutation. This case highlights TTP as an important, though uncommon, cause of young stroke and underscores the need for early recognition to enable timely management and prevent recurrent thrombotic events.

## Introduction

Thrombotic thrombocytopenic purpura (TTP) is a rare, serious form of thrombotic microangiopathy characterised by the combination of thrombocytopenia, microangiopathic hemolytic anemia, and end-organ ischemia, including neurologic and renal dysfunction [1]. TTP can be broadly classified into acquired (immune-mediated) TTP, caused by autoantibodies against ADAMTS13, and inherited or congenital TTP (Upshaw-Schulman syndrome), which results from biallelic mutations in the ADAMTS13 gene, leading to severe deficiency of ADAMTS13 activity.

It results from a deficiency of the metalloprotease ADAMTS13, leading to platelet aggregation and microvascular thrombosis, which causes red cell fragmentation and organ injury [2].

Neurological manifestations in TTP can include headaches, confusion, seizures, and ischemic stroke [3]. TTP is a rare thrombotic microangiopathy with an estimated annual incidence of approximately two to six cases per million population. Although neurologic manifestations are common, TTP is an uncommon but recognized cause of acute cerebrovascular events, particularly in young adults without conventional vascular risk factors [3,4]. Renal involvement is frequently observed in TTP and may range from mild dysfunction to renal failure due to microvascular occlusion within the kidney [5].

TTP was traditionally defined by a pentad of fever, thrombocytopenia, hemolytic anaemia, neurologic dysfunction, and renal impairment. However, contemporary practice emphasises thrombocytopenia and microangiopathic hemolysis as sufficient to suspect TTP in the appropriate clinical setting [6]. Given the high mortality associated with untreated TTP, current recommendations advocate early empiric initiation of therapy, particularly plasma exchange and corticosteroids, even before ADAMTS13 assay confirmation when clinical suspicion is high. Because ADAMTS13 assay results may not be immediately available, clinical prediction tools, such as the PLASMIC (Platelet count, combined Hemolysis parameters, Absence of active cancer, no history of solid Organ or Stem cell transplant, MCV (mean corpuscular volume), INR (international normalized ratio), and Creatinine) score, have been developed to estimate the probability of severe ADAMTS13 deficiency and help guide early therapeutic decisions.

Despite increasing awareness, TTP remains under-recognised as a cause of ischemic stroke in young adults, particularly when classical features are incomplete or when organ involvement evolves sequentially. Therefore, we report a rare case of TTP presenting as acute ischemic stroke in a young adult without conventional vascular risk factors.

## Case presentation

A 23-year-old male laboratory technician from Thiruvallur was brought by his sister with complaints of acute-onset weakness of the right upper and lower limbs and inability to speak for two days. The history obtained from the informant was reliable, and the patient had been apparently well until one month prior to presentation, when he developed sudden right-sided limb weakness accompanied by numbness. During hospital evaluation at that time, he was diagnosed with non-oliguric renal failure with increased renal parameters. The neurological symptoms cured within 24 hours without residual weakness, indicative of a transient ischemic attack.

The current episode is characterised by an acute-onset, non-progressive weakness of the right upper and lower limbs, without seizures, consciousness loss, headache, or vomiting. The patient experienced sudden inability to speak, with expressive (Broca's) aphasia. He had difficulty in lifting his right upper limb, performing fine motor activities, and difficulty in standing from sitting. There is no history of knee buckling, tripping toes, difficulty turning in bed, or bowel and bladder involvement (Figure 1).

**Figure 1:**
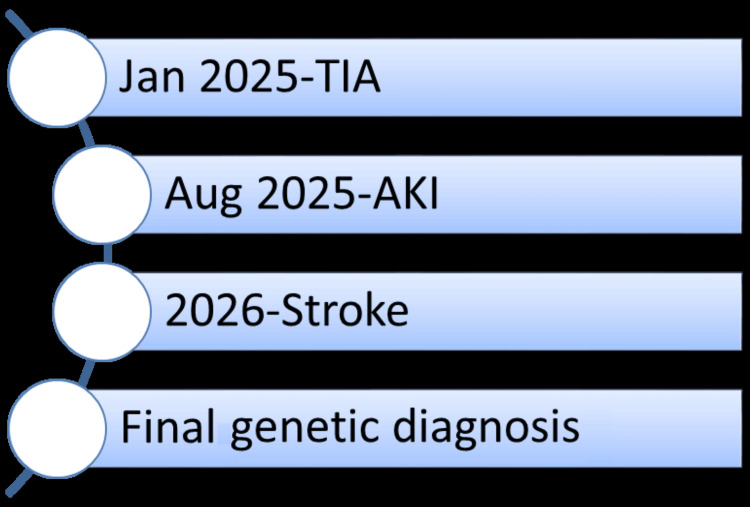
Illustrative clinical timeline This figure outlines the patient's progression from antecedent transient ischemic attacks (TIA) and renal impairment to acute ischemic stroke, laboratory evidence of thrombotic microangiopathy, confirmation of severe ADAMTS13 deficiency with genetic mutation analysis, and subsequent treatment and follow-up. Image created by authors using PowerPoint (Microsoft® Corp., Redmond, WA)

There are no symptoms of cranial nerve involvement, with visual disturbances, facial sensory loss, hearing impairment, dysphagia, dysarthria, or tongue weakness. The patient denied systemic symptoms, such as chest pain, breathlessness, fever, rash, arthralgia, or constitutional complaints.

Previously patient had a history suggestive of transient ischemic attacks in January 2025, for which medical attention was not sought. Similar episodes occurred in August 2025, during which he was found to have non-oliguric renal failure and was managed conservatively. There was no history of diabetes mellitus, hypertension, tuberculosis, congenital heart disease, thyroid disorder, seizure disorder, trauma, or previous surgeries.

He is unmarried, on a mixed diet, with normal bowel and bladder habits, and no harmful social habits. However, the family history is notable, as his elder sister died at the age of 23 due to renal failure and cerebrovascular accident, suggesting a possible inherited disorder.

Upon examination, the patient was conscious, alert, and cooperative, with a mild build and nourished presence, and exhibited pallor. There was no icterus, cyanosis, clubbing, pedal oedema, generalised lymphadenopathy, or neurocutaneous markers. Vital signs stable, with a pulse rate of 90/min, blood pressure of 130/90 mmHg, respiratory rate of 16/min, normal temperature, and saturation of 98% on room air.

Mental function assessment showed cognition and memory with Broca's aphasia. The cranial nerve test was normal. Motor inspection reported low tone and paralysis (power 0/5) of the right upper and lower limbs, with normal left side. The right plantar response was an extensor response; the left was a flexor response. Sensory examination was normal, and cerebellar functions were intact. Cardiovascular, respiratory, and abdominal examinations were ordinary. Table 1 presents the laboratory findings, and Table 2 provides the imaging findings.

**Table 1:** Laboratory findings ANA = Antinuclear antibody; Anti-dsDNA = Anti-double-stranded DNA antibody; ENA panel = Extractable nuclear antigen panel; ANCA (MPO, PR3) = Anti-neutrophil cytoplasmic antibody (myeloperoxidase, proteinase 3); APLA profile = Antiphospholipid antibody profile; Lupus anticoagulant (DRVVT) = Lupus anticoagulant (dilute Russell's viper venom time); LDH: lactate dehydrogenase

Parameter	Patient value		Units	Reference range
Hemoglobin	6.9		g/dL	13-17
Platelet count	32,000		/mcqL	150,000-400,000
LDH	734		u/L	140-280
Creatinine	8.6		mg/dL	0.9-1.3
Urea	132		mg/dL	7-20
Reticulocyte count	4.05%		-	0.5-2.5%
Urine PCR	2.38		mg/mmol	<0.2
Direct Coombs test		Negative	-	-
Complement (C3, C4)		Normal	mg/dL	C3 (80-178), C4 (15-45)
Parameter		Patient value	Units	Reference range
ANA		Negative	-	1:40-1:80
Anti-dsDNA		Negative	IU/mL	10-15
ENA Panel		Negative	-	-
ANCA (MPO, PR3)		Negative	IU/mL	-
APLA profile		Negative	U/mL	-
LUPUS anticoagulant (DRVVT)		Positive (54.9 s)	-	<1.2 s
Activated protein C resistance		Prolonged clotting time (199.5 s)	-	-
Protein C activity		85%	-	70-130%
Protein S activity		55%	-	77-143%
Antithrombin lll		126%	-	80-130%

**Table 2:** Imaging findings FLAIR: fluid attenuated inversion recovery

Parameter	Findings
Peripheral smear	Microcytic hypochromic anemia with anisopoikilocytosis and schistocytes,suggestive of microangiopathic haemolytic anemia (Figure 2)
Ultrasound abdomen	Bilateral grade 3 renal parenchymal disease
MRI brain (Figure 3)	T2/FLAIR hyperintensity in the left lentiform nucleus and body of corpus callosum with diffusion restriction and low ADC values, with acute infarct. Additional encephalomalacic and gliotic variations in left parietal and right parieto-temporal regions suggested old subacute infarcts
MRA/MRV(Figure 4)	Normal intracranial artery and venous sinuses

The presence of acute ischemic stroke, renal failure, microangiopathic hemolytic anemia, and thrombocytopenia, in the absence of alternative causes, supports a diagnosis of TTP. Table 3 presents the ADAMTS13 activity and genetic analysis.

**Table 3:** ADAMTS13 activity and genetic analysis PLASMIC = Platelet count, combined Hemolysis parameters, Absence of active cancer, no history of solid Organ or Stem cell transplant, MCV (mean corpuscular volume), INR (international normalized ratio), and Creatinine; INH ELISA = Indirect immunohistochemistry enzyme-linked immunosorbent assay

PLASMIC score	5 (Intermediate risk for severe ADAMTS13 deficiency)
ADAMTS13 activity, ADAMTS13 antibody, INH ELISA	0% 11.2 IU/Ml (0.0-15.0) - severe ADAMTS13 deficiency
Genetic analysis	Next-generation sequencing (NGS)-based clinical exome analysis identified a homozygous likely pathogenic missense variant in the ADAMTS13 gene, c.703G>C (p.Asp235His) in exon 7, consistent with hereditary thrombotic thrombocytopenic purpura

Patient treated with high-dose intravenous methylprednisolone (1 g/day) for three days, followed by oral therapy, with antiplatelets, statins, renal supportive measures, oral bicarbonate, calcium supplementation, and physiotherapy. No adverse events were observed. At discharge after completion of intravenous methylprednisolone therapy, the patient demonstrated partial neurological improvement with recovery of proximal motor function in the right lower limb and modest improvement in expressive aphasia, although significant residual weakness persisted. Hemodynamic status remained stable, platelet counts showed partial improvement, and renal dysfunction persisted, requiring continued nephrology follow-up.

The patient remains on regular follow-up with the hematology and neurology departments and continues to receive serial fresh frozen plasma (FFP) transfusions at a dose of 10-15 mL/kg every two to three weeks, with gradual clinical improvement in motor function and speech. Residual neurological deficits persisted, but the patient was ambulatory with support and able to communicate more effectively compared to initial presentation. Figures 2-4 present the peripheral smear, a representative MRI brain image demonstrating acute infarction, and magnetic resonance angiography/magnetic resonance venography (MRA/MRV) - coronal MRI sections (frontal plane) and bottom rows (MR angiography), respectively.

**Figure 2:**
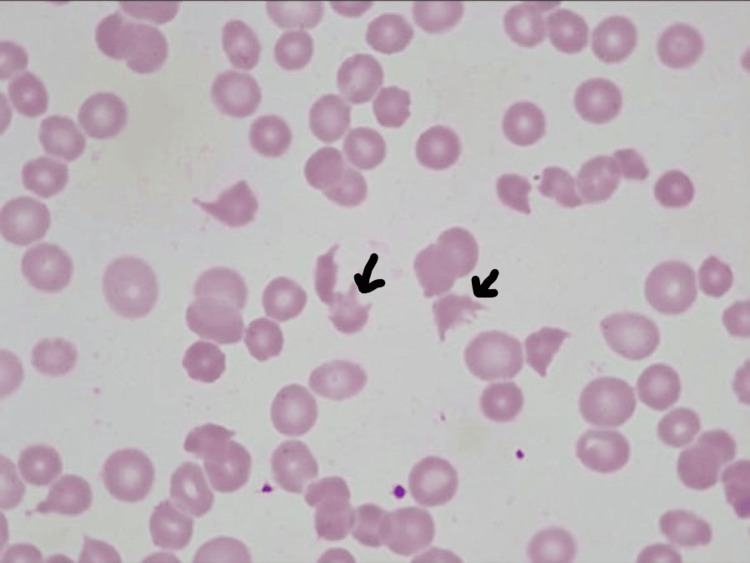
Peripheral smear The black arrow shows schistocytes (helmet cells).

**Figure 3:**
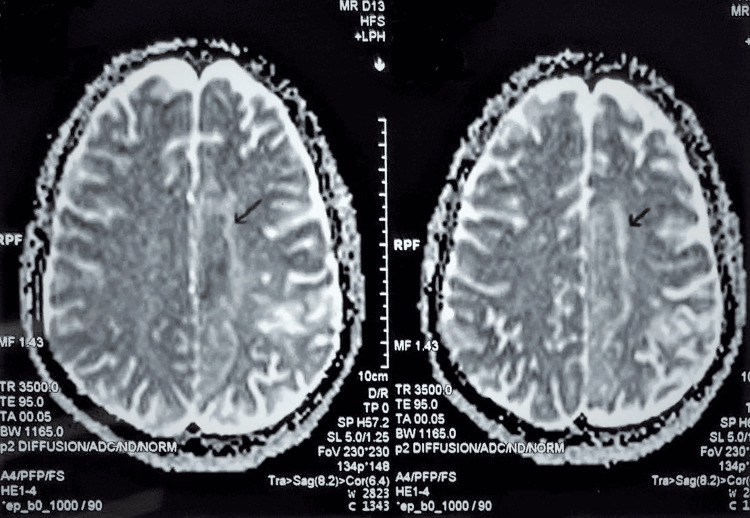
Representative MRI brain image demonstrating acute infarction Axial diffusion-weighted imaging (DWI) and T2/fluid-attenuated inversion recovery (FLAIR) sequences demonstrate areas of diffusion restriction and hyperintensity involving the left lentiform nucleus and body of the corpus callosum (black arrows), consistent with acute infarction. Additional chronic encephalomalacic and gliotic changes in the left parietal and right parieto-temporal regions are suggestive of prior ischemic insult.

**Figure 4:**
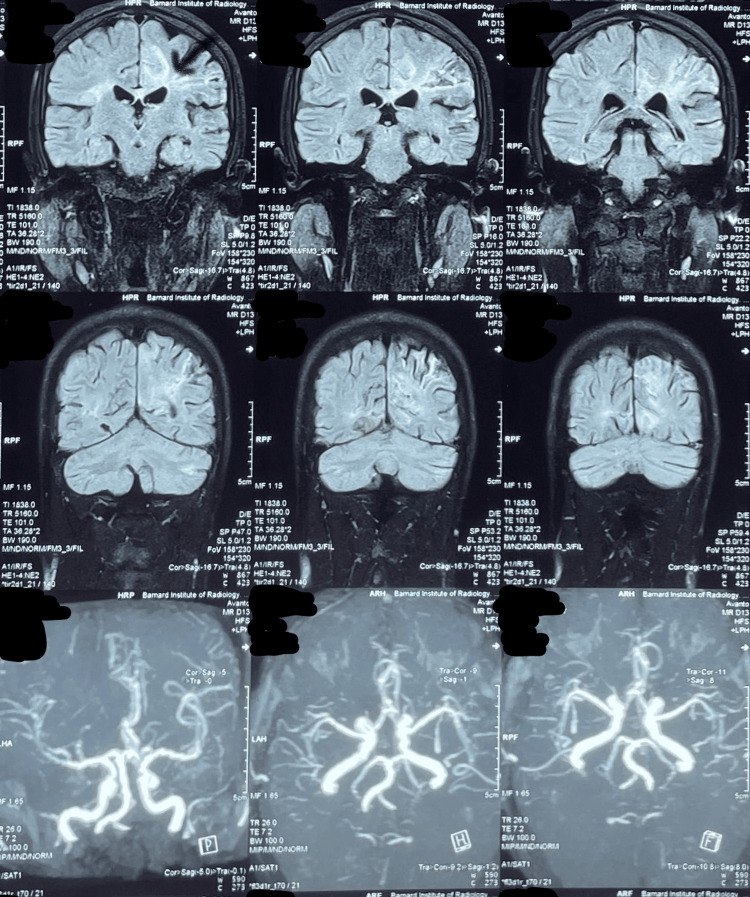
MRA/MRV - coronal MRI sections (frontal plane), bottom rows: MR angiography demonstrating normal intracranial arterial circulation with no evidence of large-vessel occlusion, stenosis, or venous thrombosis

## Discussion

TTP is a rare thrombotic microangiopathy identified by a pentad of thrombocytopenia, microangiopathic hemolytic anemia, renal dysfunction, neurological involvement, and fever. It results from severely reduced activity of the von Willebrand factor-cleaving protease ADAMTS13, leading to accumulation of ultra-large von Willebrand factor multimers and widespread platelet aggregation in small vessels [2,3,7]. In adults, TTP typically presents with fever and multiorgan involvement; however, presentations can vary widely, and isolated neurologic or renal symptoms may predominate [3,8]. Figure 5 presents the schematic representation of the pathophysiology of TTP.

**Figure 5:**
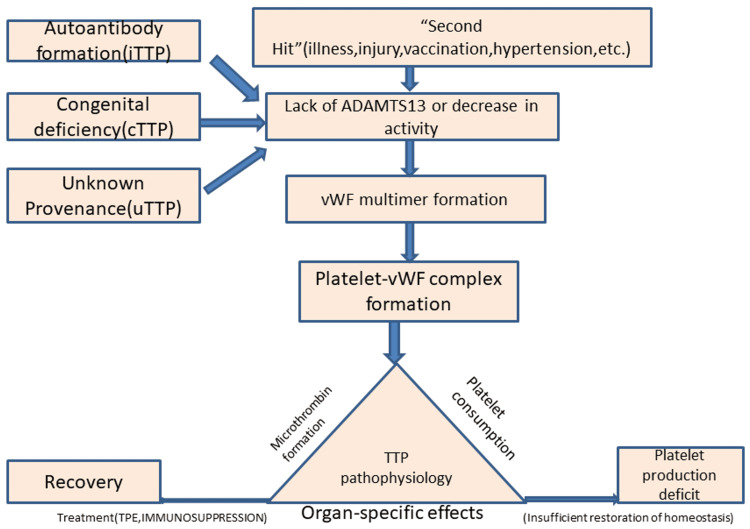
Schematic representation of the pathophysiology of congenital thrombotic thrombocytopenic purpura demonstrating ADAMTS13 deficiency leading to accumulation of ultra-large von Willebrand factor multimers, resulting in platelet aggregation, microvascular thrombosis, and end-organ ischemia In normal physiology, ADAMTS13 cleaves ultra-large von Willebrand factor (vWF) multimers into smaller units, preventing excessive platelet aggregation. In ADAMTS13 deficiency, ultra-large vWF multimers accumulate, leading to platelet aggregation, microvascular thrombosis, and red blood cell fragmentation. The figure was created by the authors using Microsoft PowerPoint based on established pathophysiological concepts without the use of generative artificial intelligence.

Neurological manifestations in TTP are common and can range from confusion and headache to seizures and focal deficits, including stroke [8]. Ischemic stroke in young adults, particularly those without conventional vascular risk factors, should prompt consideration of systemic thrombotic and microangiopathic causes, including TTP [8]. In the present case, the patient presented with an acute onset of right hemiplegia and expressive aphasia along with antecedent transient ischemic attacks, underscoring the importance of recognising TTP as a cause of recurrent cerebrovascular events in young patients [4]. The absence of large-vessel occlusion, stenosis, or cerebral venous thrombosis on MRA/MRV further supported a microvascular thrombotic mechanism of stroke, consistent with the platelet-rich small-vessel thrombi characteristic of TTP. Similar to previously reported cases, our patient lacked conventional vascular risk factors but differed in having genetically confirmed congenital TTP.

Renal involvement in TTP often reflects thrombotic microangiopathy affecting the renal microvasculature and may manifest as elevated creatinine, proteinuria, or hematuria [10]. In our patient, severe renal failure and an elevated urine protein-creatinine ratio supported significant renal involvement. Although antiphospholipid antibody syndrome can present with combined neurologic and renal thrombosis, this was less likely here, given negative anticardiolipin and β2-glycoprotein antibodies, with only lupus anticoagulant positivity and no clinical features of systemic lupus erythematosus [2,11].

The PLASMIC score is a validated clinical tool to estimate the probability of severe ADAMTS13 deficiency in suspected TTP and helps guide early therapeutic decisions when ADAMTS13 assay results are delayed [12]. A score of 5 in our patient indicated intermediate risk. Next-generation sequencing (NGS)-based clinical exome analysis subsequently identified a homozygous likely pathogenic missense variant in the ADAMTS13 gene, c.703G>C (p.Asp235His) in exon 7, confirming congenital TTP. The variant was classified as likely pathogenic according to the American College of Medical Genetics and Genomics (ACMG) criteria by the reporting molecular laboratory based on its rarity, predicted deleterious functional effect, evolutionary conservation, and the associated phenotype of severe ADAMTS13 deficiency. Pathogenic missense variants in ADAMTS13 have previously been associated with recurrent thrombotic microangiopathic events, ischemic stroke, and renal involvement in congenital TTP, although genotype-phenotype correlations remain incompletely defined because of the rarity of the disorder. Although hereditary TTP follows an autosomal recessive inheritance pattern, the absence of symptoms in the patient's sibling does not exclude hereditary transmission because of variable clinical expression. Genetic testing of the patient's parents and other family members could not be performed because of logistical and financial limitations. Genetic forms of TTP are rare but should be considered in young patients with recurrent microangiopathic events and a suggestive family history.

Quick immunosuppressive therapy with corticosteroids and plasma exchange has been shown to improve outcomes in immune-mediated TTP by reducing autoantibody production and replenishing ADAMTS13 activity [13,14]. In congenital TTP, plasma infusion and careful supportive management remain the mainstays of therapy. Follow-up neuroimaging could not be obtained during the preparation of this report, which limited radiological assessment of treatment response and long-term progression.

This case highlights the need for early recognition and comprehensive evaluation of young stroke patients with thrombocytopenia and hemolysis to identify underlying thrombotic microangiopathy. Recognising early using clinical features, laboratory tests, and tools such as PLASMIC score is important; early diagnosis and precise treatment can improve outcomes and prevent thrombotic events [12,14,15].

## Conclusions

Congenital TTP is a rare but life-threatening condition that may present with recurrent ischemic strokes in young individuals without conventional vascular risk factors. Early recognition based on clinical suspicion, laboratory evidence of microangiopathic hemolytic anemia, and supportive scoring systems such as the PLASMIC score is crucial. Genetic confirmation of ADAMTS13 deficiency establishes the diagnosis and guides long-term management. This case highlights the importance of considering thrombotic microangiopathy in atypical stroke presentations to enable timely intervention and prevent recurrent morbidity.
